# Investigating How Thbs4 Regulates Degeneration and Regeneration of the Peripheral Nerve

**DOI:** 10.3390/biomedicines13102375

**Published:** 2025-09-28

**Authors:** Yi Yao, Yiyue Zhou, Zixu Zhang, Yuxiao Huang, Taoran Jiang, Yiming Xia, Dandan Gu, Xi Gu, Huiyuan Bai, Maorong Jiang, Chunmei Yu

**Affiliations:** 1 School of Public Health, Nantong University, Nantong 226019, China; 2 School of Life Sciences, Key Laboratory of Neuroregeneration of Jiangsu and Ministry of Education, Co-Innovation Center of Neuroregeneration, Nantong University, Nantong 226019, China; 3 Medical School, Nantong University, Nantong 226001, China

**Keywords:** Thbs4, nerve injury repair, neurons, axonal regeneration, neurological functional recovery

## Abstract

**Objective:** Molecular biology techniques were employed to investigate the effects of thrombospondin-4 (Thbs4) expression in dorsal root ganglion (DRG) on peripheral nerve injury repair and regeneration, as well as to elucidate the underlying molecular mechanisms. **Methods:** A sciatic nerve transection model in rat was established to analyze Thbs4 expression and localization in DRG tissues after injury. Both siRNA and adeno-associated virus (AAV) were used to knockdown or overexpress Thbs4. The effects of knockdown and overexpression of Thbs4 on axon growth were assessed using immunofluorescence staining. The roles of Thbs4 in peripheral nerve injury repair and regeneration were determined using behavioral assays, electrophysiological recordings, and transmission electron microscopy. **Results:** Thbs4 was primarily localized in the cell membrane and cytoplasm of DRG neurons but was also found in the intercellular spaces. In vitro experiments demonstrated that Thbs4 overexpression promoted axonal regeneration and reduced neuronal apoptosis. They also showed that Thbs4 overexpression accelerated sciatic nerve regeneration and enhanced the recovery of motor and sensory functions. Conversely, Thbs4 knockdown had the opposite effects. This study also showed that the knockdown or overexpression of Thbs4 significantly altered the expression of NF-κB and ERK signaling pathways, suggesting their involvement in peripheral nerve repair and regeneration. **Conclusions:** Thbs4 expression in DRG tissues is significantly altered following sciatic nerve injury. The NF-κB and ERK may be involved in regulating the repair and regeneration of the peripheral nerve by Thbs4.

## 1. Introduction

Peripheral nerve injury refers to damage to nerves outside the central nervous system. It often results in sensory disturbance, motor impairments, nutritional imbalances and other symptoms in the affected areas, posing a significant challenge for regenerative medicine [1,2]. After peripheral nerve injury, the interruption of retrograde transport and loss of nutrient supply can lead to neuronal cell death [2,3,4]. Additionally, the existence of gaps can prevent the growth of proximal axons from extending into the distal end, causing scar formation that blocks axonal regeneration [5,6]. Natural regeneration following peripheral nerve injury is usually requires surgery to repair [7]. Although the peripheral nervous system has some regenerative capacity, complete functional recovery to pre-injury levels remains elusive [8]. There have been previous studies of peripheral nerve injury repair, but the nerves complex anatomical structure and physiological function and the biology effects of injury repair have hindered breakthroughs in related research [9]. Currently, promoting rapid nerve repair after injury is a hot topic in worldwide research [7,10].

Thrombospondin-4 (Thbs4) is a member of the extracellular, multidomain, oligomeric, and calcium-binding glycoprotein family [11]. Thbs4 was initially identified in the African clawed frog genome, and primarily regulates various cell–cell interactions [12,13]. The family can be divided into two subgroups based on structural and functional domain similarities: Group A (TSP1/2) and Group B (TSP3/4/5) [14,15]. Thbs4, the fourth member of the thrombospondin family, has been shown to be expressed in many cells and tissues, including cardiomyocytes and nerve tissues [14,16]. In disease contexts, Thbs4 is involved in several tumor-related diseases and mainly expressed in the extracellular matrix surrounding tumor cells, particularly in regions of high cell density and invasiveness [11,17]. In addition, Thbs4 plays a crucial role in protecting the heart from pathological remodeling [18].

Studies have shown that in the nervous system, neuropathic pain caused by peripheral nerve injury was associated with the upregulation of Thbs4 expression, and Thbs4 could mediate intracellular Ca^2+^ signaling in peripheral sensory neurons. It may thus be a target for developing analgesic drugs for neuropathic pain [19,20,21]. During nervous system development, Thbs4 has been found to be an attractive substrate for certain neuronal processes, playing an important role in neurite outgrowth [22]. It has been shown to induce synapse formation by interacting with the neuronal receptor calcium channel α2δ1 subunit protein, making it a key molecule in axon growth, as demonstrated in cultures of rat retinal ganglion cells (RGCs) [23].

In addition, it was reported that Thbs4 promoted neuronal differentiation of neuron glial antigen 2 (NG2) glial cells. Previous research has provided much evidence for Thbs4s in playing a crucial role in neurodevelopmental biology. In our previous research, we used gene chip and protein chip technologies to analyze changes in gene and protein expression during Wallerian degeneration (WD) after sciatic nerve injury in rats. It was found that WD was regulated by multiple core molecules, including Fas, FasL, BIRC3, and Thbs4, although the functions of most genes remained unclear [24]. Although Thbs4 was expressed in various regions of the nervous system, its role remains unclear. We assumed that Thbs4 played an important role in peripheral nerve injury and repair. Therefore, in this study, we investigated the time-course expression of Thbs4 during peripheral nerve regeneration and studied its biological functions and mechanisms in regulating the degeneration and regeneration of peripheral nerve. Our findings will thus provide the experimental and theoretical basis underlying Thbs4’s role in nerve regeneration and functional recovery after peripheral nerve injury.

## 2. Methods

### 2.1. Establishment of Sciatic Nerve Transection Model in Rats

The male Sprague–Dawley (SD) rat (8 weeks of age) weighing 200 ± 20 g was obtained from the Laboratory Animal Center of Nantong University, and kept in a room with constant temperature (22 ± 2 °C) and humidity, and a light/dark (12:12 h) cycle. This study was approved by the Institutional Animal Care and Use Committee of Nantong University (approval No. 2019-nsfc004) on 1 March 2019. The animal models were developed according to the guide for the Care and Use of Laboratory Animals. All experiments were designed and reported according to the Animal Research: Reporting of In Vivo Experiments (ARRIVE) guidelines [25].

The sciatic nerve transection model of rat was established as we have previously reported [26]. Briefly, rats were anesthetized via an intraperitoneal injection with a compound anesthetic (10 mg/kg xylazine, 95 mg/kg ketamine, 0.7 mg/kg acepromazine) at a dose of 0.3 mL/100 g (body weight). The hair on the left hind limb was shaved, and the skin was disinfected with alcohol and iodine. The sciatic nerve was exposed by blunt dissection and transected with a 0.5 cm gap, and the wound was sutured and disinfected with iodophor. The rats were then placed in a cage to recover and transferred to a controlled environment laboratory for care after awakening.

### 2.2. Quantitative RT-PCR (qRT-PCR) and Western Blot

qRT-PCR and a Western blot were used to detect the time-course expressions of Thbs4 in dorsal root ganglia (DRG) tissues after sciatic nerve transection. Total RNA and protein were extracted from L4 and L5 DRG tissues at 2, 4, 7, 14, 21 and 28 days after transection. The total RNA was reverse-transcribed into first-strand cDNA, and the analysis was performed using PCR. The primer sequences used are listed in Table 1. The concentration of extracted protein was determined using a BCA kit (Beyotime Biotechnology, Shanghai, China), and the protein was separated via SDS-PAGE gel electrophoresis. Primary anti-Thbs4 antibody was used to detect expression after being transferred to the PVDF membrane. The antibodies used in this study are listed in Table 2. ImageJ software (1.54g) (NIH, Bethesda, MD, USA) was applied to quantify the target signal.

### 2.3. Immunofluorescence (IF) Staining

The L4 and L5 DRG tissues at 2, 4, 7, 14, 21, and 28 days after transection were fixed with 4% paraformaldehyde (PFA) overnight at 4 °C, and then embedded in OCT (Sakura Finetek, Torrance, CA). The embedded DRG tissues were cut into 12 μm slices and incubated with primary anti-Thbs4 and anti-Tuj1 antibodies overnight at 4 °C. Then the slices were further incubated with second antibodies then stained with DAPI. The primary antibodies and second antibodies are also listed in Table 2. After staining, the slices were observed using ZEISS Axio Scope.A1 microscope (Oberkochen, Germany).

### 2.4. Primary Cultured DRG Neuron Cells and Transfection

As described in our previous report [27], DRGs were removed from adult rats then digested using collagenase for 90 min and trypsin for 10 min at 37 °C. A subsequent cell suspension was cultured in the poly-L-lysine pre-coated plate wells after being triturated and centrifuged. The small interfering RNAs (siRNAs) used for knockdown and negative control siRNA (scramble) were obtained from RiboBio (Guangzhou, China), and the adenovirus-associated virus (AAV) used for overexpression and negative control (empty vector) were obtained from Genechem (Shanghai, China). The siRNA target sequences are listed in Table 3. Using the instructions provided by the manufacturer, siRNA, RNA iMAX and Opti-MEM were mixed for 15 min at room temperature. Then, the mix was added onto the plate well, incubated for 16 h, and finally media were replaced with neuron culture medium. For AAV transfection, after the DRG neuron cells were cultured in neuronal medium for 1 day, the cells were infected with the AAV for 16 h, after which media were then replaced with neuronal medium.

### 2.5. CCK8 Assay, TUNEL and IF Staining

The CCK8 assay, TUNEL, and IF staining were adapted to measure the effects of Thbs4 on the cell viability, apoptosis, and neurite outgrowth of DRG neuron cells. After the DRG neuron cells were transfected, the CCK8 (Vazyme, Nanjing, China) was added into the culture media for 2 h. A microplate reader (bio-tek, Agilent Technologies, Santa Clara, CA, USA) was used at a 450 nm wavelength. After transfection, the DRG neuron cells were fixed, and TUNEL or IF staining was performed with anti-Tuj1 antibody. The ImageJ software (1.54g) (NIH, Bethesda, MD, USA) was used to quantify the neurite outgrowth length.

### 2.6. Intrathecal Injection of AAV

AAV injection was employed in in vivo models to knockdown or overexpress Thbs4. After complete anesthesia, the L4-L6 vertebrae of rat were exposed along the midline of the back, and the intervertebral foramen was uncovered. Microsyringes with electrode tips were used to inject AAV solutions into the intrathecal space. The injection volumes were as follows: knockdown Thbs4 negative control group (NC-sh), 2 μL AAV + 8 μL PBS; knockdown Thbs4 group (AAV-sh-Thbs4), 4.2 μL AAV + 5.8 μL PBS; overexpression Thbs4 negative control group (NC-OE), 2 μL AAV + 8 μL PBS; overexpression Thbs4 group (OE-Thbs4), 8 μL AAV + 2 μL PBS. After injection, the wound was sutured and disinfected with iodophor. The rats were then placed in a cage and transferred to a controlled environment laboratory for feeding after awakening. The sciatic nerve transection model was established 14 days post-injection. Following transection with a 0.5 cm gap, a 0.7 cm silicone tube (Invitrogen, New York, NJ, USA) was sutured between the two stump ends.

### 2.7. Hematoxylin and Eosin (H&E), and IF Staining

After anesthesia, rats were perfused with normal saline and 4% PFA. After the regenerated sciatic nerve was stripped from the silicone tube, the sciatic nerve and gastrocnemius muscle were collected and post-fixed with 4% PFA overnight at 4 °C. The gastrocnemius muscle was weighed to calculate the muscle wet weight ratio before post-fixation. The fixed regenerated sciatic nerve and gastrocnemius muscle were embedded in paraffin and cut into 5 μm thick sections. H&E staining was performed on the sections of gastrocnemius muscle, photographs were taken from three random fields and analyzed with a Leica QWin software package to measure the cross-sectional area (CSA) of the muscle fibers. The sections of the regenerated sciatic nerve were IF-stained with anti-SCG10 antibody.

### 2.8. Transmission Electron Microscope (TEM)

The effects of Thbs4 on the regenerated myelin sheath were observed with a TEM. The regenerated sciatic nerve was fixed with a pre-cooled glutaraldehyde, and then the solution was replaced with 1% osmium acid for post-fixation. The sciatic nerve was cut into ultra-thin sections and stained with lead citrate and uranyl acetate. The regenerated myelin sheath was observed under the TEM (JEOL Ltd., Tokyo, Japan).

### 2.9. Behavioral Experiment

Behavioral tests were performed at 2, 4, 6 and 8 weeks after transection. In this study, behavioral tests included footprint analysis and the assessment of thermal hyperalgesia and mechanical allodynia. During the behavioral tests, researchers were blinded to grouping.

#### 2.9.1. Footprint Analysis

A footprint analysis of rats was performed at 2, 4, 6, and 8 weeks after sciatic nerve transection using the Catwalk gait analysis system (Noldus, Leesburg, VA, USA). The footprints were recorded using a Catwalk fluorescent plate as rats walked on the runway. The print length (PL), the toe spread (TS), and the intermediary toe spread (IT) of non-operated (N) and experimental (E) hind legs were obtained. The sciatic function index (SFI) was calculated using the following formula [26]: SFI = 109.5 (ETS − NTS)/NTS − 38.3 (EPL − NPL)/NPL + 13.3 (EIT − NIT)/NIT − 8.8.

#### 2.9.2. Assessment of Thermal Hyperalgesia and Mechanical Allodynia

The rats, at 2, 4, 6, and 8 weeks after transection, were subjected to tests to assess thermal hyperalgesia and mechanical allodynia. All rats were fully familiarized with the testing environment at least two days before measurement. In brief, rats were placed in a compartment of a heat insulation board for 5–10 min to adapt to the environment, and then the middle part of the plantar of the injured hind limbs was thermally stimulated by a constant light source. The time from the start of the thermal stimulation to the occurrence of foot retraction was recorded. The withdrawal time was recorded at least three times, during an interval of about 30 min. The 30 s cut-off time was used to avoid plantar skin damage.

The Aesthesio Von Frey Hairs tool (Yuyan Instruments, Shanghai, China) was used to measure mechanical allodynia. The rats were placed in the compartment to habituate to the environment 1 h before testing. The injured plantar was stimulated with a series of filaments of different diameters, and the paw withdrawal thresholds were recorded. Each rat was tested at least three times, and all data were recorded for subsequent statistical analysis.

### 2.10. Electrophysiological Test

At 8 weeks after sciatic nerve transection, electrophysiological equipment was used to record compound muscle action potential (CMAP) by inserting the recording electrode into the gastrocnemius muscle and stimulating electrodes in the proximal or distal sciatic nerve.

### 2.11. Statistical Analysis

All data were expressed as mean ± standard deviation (SD), and analyzed using GraphPad Prism software (version 8) (GraphPad, La Jolla, CA, USA). One-way ANOVA with subsequent Turkey’s tests was applied to analyze statistical differences between groups. A significance level of *p* < 0.05 was considered statistically significant.

## 3. Results

### 3.1. Time-Course Expressions of Thbs4 in DRG After Sciatic Nerve Transection

**Figure 1:**
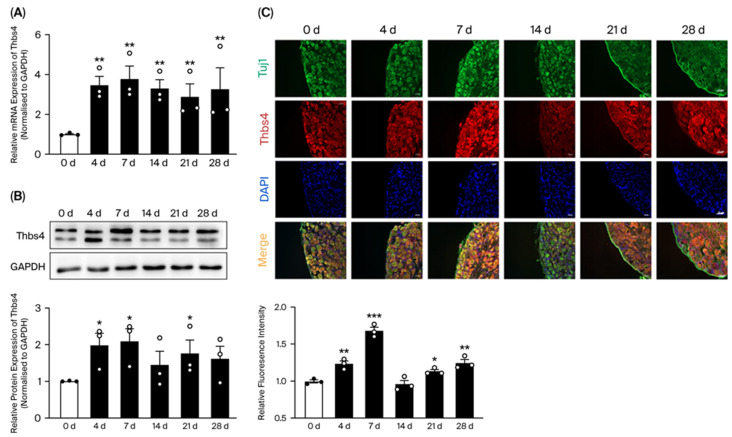
Time-course expressions of Thbs4 in DRG tissue after sciatic nerve transection in rats. (**A**) qRT-PCR was used to detect time-course mRNA expressions of Thbs4. (**B**) Western blot was used to detect time-course expressions of Thbs4. (**C**) Immunofluorescence staining of Thbs4 in DRG tissues at different time points after sciatic nerve transection. Red: Thbs4, green: Tuj1, blue: DAPI, Bar = 50 μm; GAPDH as internal control, 0 day as control group, *n* = 3. Versus 0 d, * *p* < 0.05, ** *p* < 0.01 and *** *p* < 0.001. Thbs4, thrombospondin-4. qRT-PCR, real-time quantitative PCR. DRG, dorsal root ganglia. GAPDH, glyceraldehyde-3-phosphate dehydrogenase. Tuj1, beta III Tubulin.

qRT-PCR/Western blot was used to measure time-course expressions of Thbs4. The results showed that Thbs4 expressions were increased in DRG tissues after sciatic nerve injury, with the highest expression observed on day 7 (Figure 1A,B). This indicated that Thbs4 might play a significant role in regeneration following sciatic nerve injury. IF staining was employed to further measure the expression of Thbs4 in DRG (Figure 1C). The results of relative fluorescence intensity similarly showed that Thbs4 expression reached its peak in DRG tissues on day 7 after transection.

### 3.2. Thbs4 Localization in DRG Tissues and Neuronal Cells

**Figure 2:**
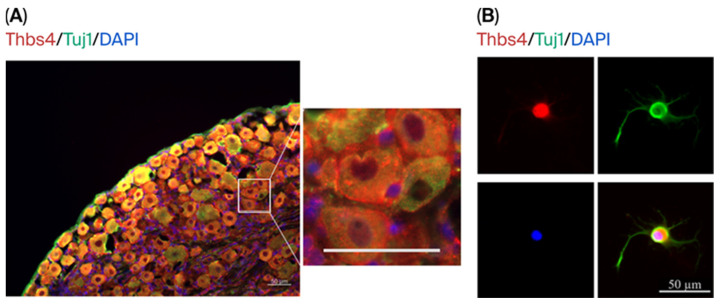
Localization of Thbs4 in DRG tissues and cells. (**A**) Immunofluorescence staining was used to detect Thbs4 expression localization. Thbs4 localization in DRG tissue is shown on the left and partial enlargement on the right. (**B**) Thbs4 localization in DRG neuron cells. Red: Thbs4, green: Tuj1, blue: DAPI. Bar = 50 μm. Thbs4, thrombospondin-4. Tuj1, beta III Tubulin.

The localization was detected via the IF staining of Thbs4 and Tuj1 in DRG tissues and neuronal cells. The staining results showed that the positive signal of Thbs4 was distributed in both the cytoplasmic stroma and intercellular spaces of DRG tissues (Figure 2A). We performed IF staining to further detect the localization of Thbs4 in primary cultured DRG neurons cells. The subcellular localization of Thbs4 was mainly distributed in the DRG neuronal cytoplasm (Figure 2B).

### 3.3. Effects of Thbs4 on Viability, Neurite Outgrowth and Apoptosis of DRG Neurons

**Figure 3:**
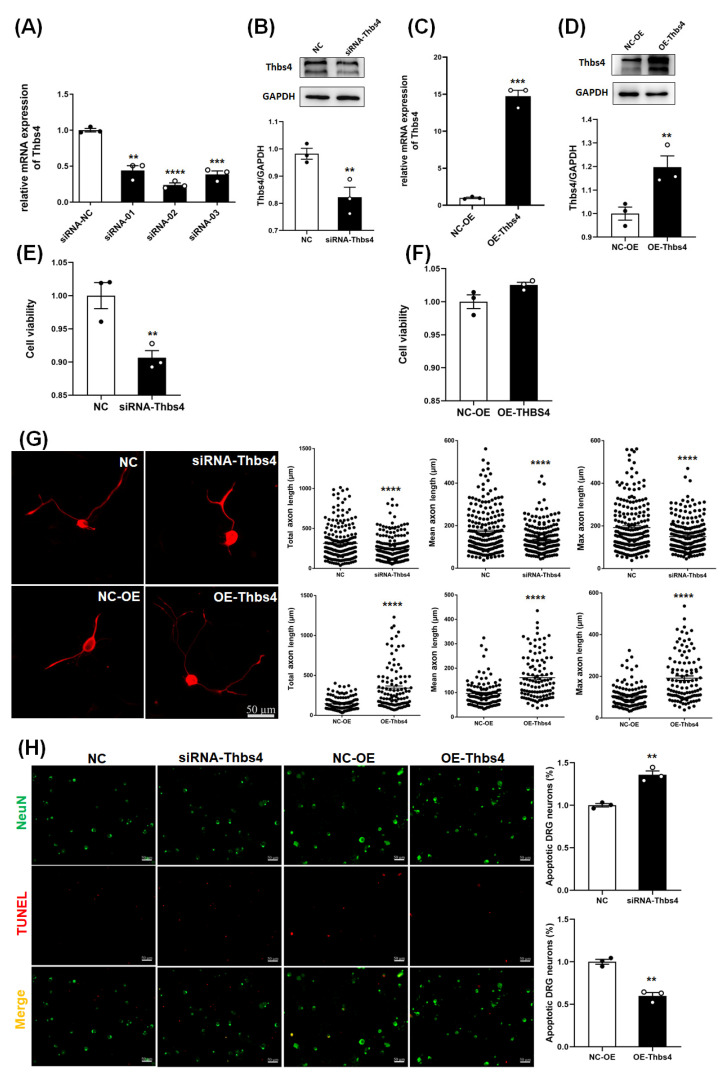
Effects of in vitro knockdown or overexpression of Thbs4 on viability, neurite outgrowth and apoptosis of DRG neuron cells. (**A**) qRT-PCR was used to detect mRNA expressions of Thbs4 after siRNA transfection. (**B**) Western blot was used to detect expressions of Thbs4 after siRNA-02 transfection. (**C**) qRT-PCR and (**D**) Western blot was used to detect expressions of Thbs4 after AAV transfection. (**E**,**F**) CCK8 assay was used to measure the effects of Thbs4 knockdown or overexpression on DRG neuronal cell viability. (**G**) Immunofluorescence staining of Tuj1 was used to assess the effects of Thbs4 on neurite outgrowth of DRG neuron cells. Red: Tuj1, Bar = 50 μm. (**H**) TUNEL staining was used to analyze the effects of Thbs4 on apoptosis of DRG neuron cell. Bar = 50 μm. GADPH as internal control. NC or NC-OE was used as negative control. *n* = 3, versus negative control, ** *p* < 0.01, *** *p* < 0.001, and **** *p* < 0.0001. Thbs4, thrombospondin-4. GAPDH, glyceraldehyde-3-phosphate dehydrogenase. Tuj1, beta III Tubulin. qRT-PCR, real-time quantitative PCR. DRG, dorsal root ganglia. siRNA, small interfering RNA. NC, negative control of siRNA. NC-OE, negative control of overexpression. AAV, adeno-associated virus. NeuN, neuronal nuclei antigen.

To further investigate the effects of Thbs4 on DRG neuron cells, we knocked down Thbs4 expression via siRNA transfection, and overexpressed Thbs4 via AAV infection. qRT-PCR was applied to analyze the expressions of Thbs4 after transfection with three siRNAs (siRNA-01, 02 and 03). The mRNA expressions were all significantly decreased; however, expression was lowest in cells transfected with siRNA-02 (Figure 3A). Therefore, siRNA-02 was used for in vitro knockdown in the next experiments. A Western blot was used to detect the expression of Thbs4 after it was transfected with siRNA-02. As expected, the expression of Thbs4 was dramatically reduced after transfection (Figure 3B). The expressions of Thbs4 markedly increased after transfection with AAV (Figure 3C,D).

A CCK8 assay was applied to measure the viability of DRG neurons after the knockdown or overexpression of Thbs4. The results showed that cell viability significantly declined after knockdown (Figure 3E). However, the cell viability was not obviously lower after overexpression (Figure 3F). This perhaps shows that Thbs4 plays an important role in promoting neurite outgrowth.

IF staining was used to investigate the effect of Thbs4 on neurite outgrowth. The total axon length, mean axon length and max axon length were determined through statistical analysis. In the Thbs4 knockdown group, the total axon length, mean axon length and max axon length were all significantly reduced. However, the axon length was reasonably increased in the overexpression group (Figure 3G). This result indicates that Thbs4 promotes neurite outgrowth.

The effect of Thbs4 on DRG neuronal apoptosis was measured via TUNEL staining. The relative apoptosis rate of DRG neurons was significantly higher in the knockdown group, while the apoptosis rate was lower in the overexpression group (Figure 3H). This indicates that Thbs4 might promote the survival of DRG neuron cells.

### 3.4. Effects of Thbs4 on Growth and Apoptosis-Related Factors in DRG Neurons

**Figure 4:**
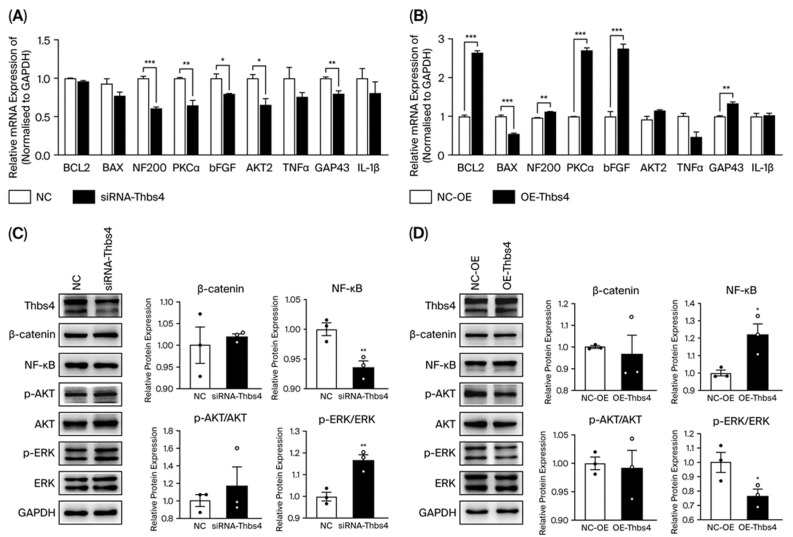
Effects of knockdown or overexpression of Thbs4 on related factors in DRG neuron cells. Before (**A**) and after (**B**) knockdown or overexpression of Thbs4, qRT-PCR was used to detect the expressions of growth- and apoptosis-related factors in DRG neuron cells. Before (**C**) and after (**D**) knockdown or overexpression of Thbs4, Western blot was used to detect the expressions of growth factors and signal pathway factors. NC or NC-OE was used as negative controls. *n* = 3, versus negative control, * *p* < 0.05, ** *p* < 0.01 and *** *p* < 0.001. siRNA, small interfering RNA. NC, negative control of siRNA. AAV, adeno-associated virus. NC-OE, negative control of overexpressed AAV. Bcl2, B-cell lymphoma-2. Bax, Bcl-associated X protein. NF200, neurofilament 200. PKCα, protein kinase Cα. bFGF, basic fibroblast growth factor. AKT2, threonine Kinase 2. TNFα, tumor necrosis factor α. GAP43, growth associated protein 43. IL-1β, interleukine-1β. Thbs4, thrombospondin-4. NF-κB, nuclear factor-κB. ERK, extracellular regulated protein kinase. GAPDH, glyceraldehyde-3-phosphate dehydrogenase. DRG, dorsal root ganglion. qRT-PCR, real-time quantitative PCR.

To explore how Thbs4 regulates biological functions, we selected factors closely associated with apoptosis, axonal growth, and pro-inflammatory factors. qRT-PCR analysis showed that the expressions of NF200, PKCα, bFGF and GAP43 were all significantly decreased after Thbs4 knockdown, and the reverse was observed after Thbs4 overexpression, indicating that Thbs4 had a positive regulatory effect on DRG neuron axon growth and cell survival (Figure 4A,B). Neither the mRNA expression of Bcl2 or that of Bax changed significantly after Thbs4 knockdown; however, Bcl2 expression significantly increased, and that of Bax decreased after Thbs4 overexpression, as observed through qRT-PCR analysis. Western blot was applied to detect pathway proteins after Thbs4 knockdown or overexpression. The results showed that NF-κB was significantly downregulated, while p-ERK/ERK was significantly upregulated in the Thbs4 knockdown group, compared with control group (Figure 4C). However, Thbs4 overexpression led to NF-κB’s upregulation and p-ERK/ERK’s downregulation (Figure 4D).

### 3.5. Effects of Thbs4 on Sciatic Nerve Regeneration In Vivo

**Figure 5:**
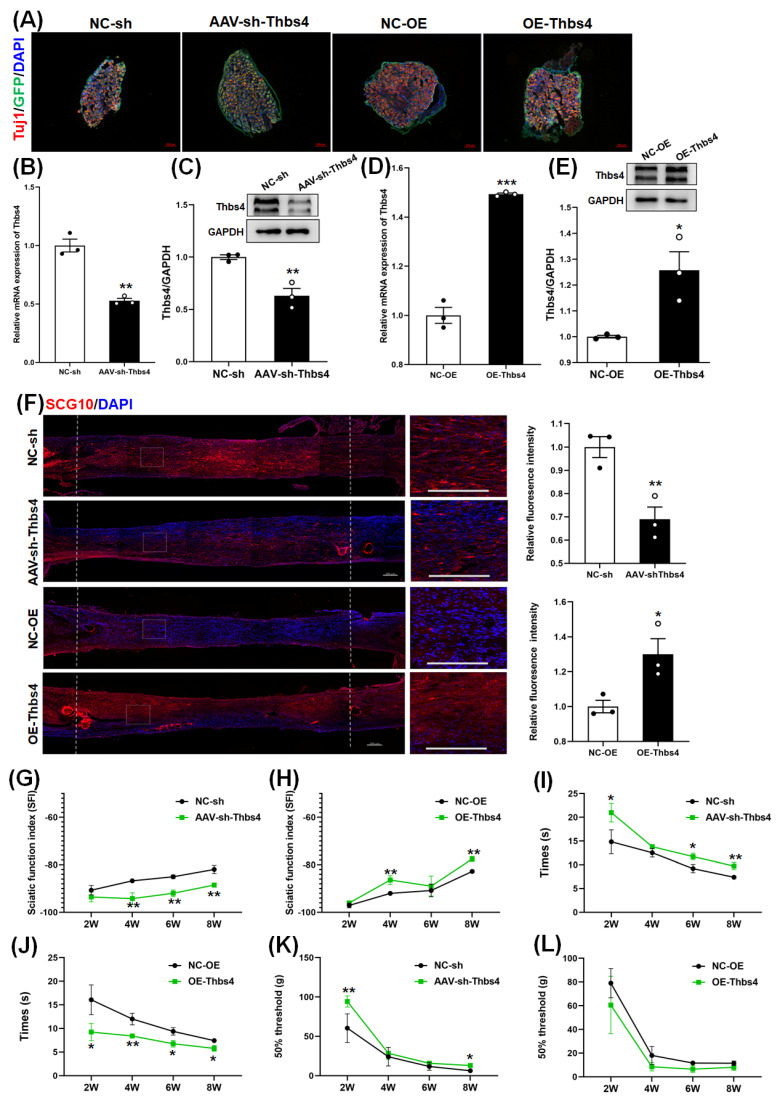
Effects of in vivo knockdown or overexpression of Thbs4 on sciatic nerve regeneration. (**A**) Immunofluorescence staining of Tuj1 after intrathecal injection with AAV. (**B**,**D**) qRT-PCR and (**C**,**E**) Western blot after intrathecal injection for 7 days was applied to detect the expression of Thbs4. (**F**) SCG10 immunofluorescence staining of sciatic nerve after knockdown or overexpression of Thbs4 for 2 weeks. red: SCG10, blue: DAPI, Bar = 200 μm. (**G**,**H**) show the sciatic nerve function index (SFI) of rats after knockdown and overexpression of Thbs4, respectively, at 2 weeks, 4 weeks, 6 weeks, and 8 weeks. (**I**,**J**) show the withdrawal time after light stimulation at 2 weeks, 4 weeks, 6 weeks, and 8 weeks after knockdown or overexpression of Thbs4, respectively. (**K**,**L**) show the withdrawal threshold after mechanical pain stimulation at 2 weeks, 4 weeks, 6 weeks, and 8 weeks after knockdown and overexpression of Thbs4, respectively. NC-sh or NC-OE was used as negative controls. *n* = 3, versus negative control, * *p* < 0.05, ** *p* < 0.01 and *** *p* < 0.001. sh, short hairpin RNA. NC-sh, negative control of shRNA. AAV, adeno-associated virus. NC-OE, negative control of overexpressed AAV. Thbs4, thrombospondin-4. GAPDH, glyceraldehyde-3-phosphate dehydrogenase. SCG10, superior cervical ganglion 10.

In the sciatic nerve regeneration model, rats were intrathecally injected with AAV to knockdown or overexpress Thbs4. Because the GFP gene was included in the AAV virus recombinant vector. the positive signals of Tuj1 and GFP could be observed in DRG after AAV injection and IF staining, (Figure 5A). This indicated that injection with AAV could knockdown or overexpress Thbs4. Further, qRT-PCR and a Western blot were used to verify the knockdown or expression of Thbs4 after injection. The results showed that intrathecal injection with AAV could knockdown or overexpress Thbs4 in DRG tissues (Figure 5B–E).

The sciatic nerve transection model was established 14 days after intrathecal injection with AAV, and the stump ends were sutured with a 0.7 cm silicone tube. After sciatic nerve transection for 2 weeks, the nerves were isolated from silicone tubes, and IF staining with anti-SCG10 antibody was performed (Figure 5F). The fluorescence intensity of SCG10 in the knockdown group was weaker than that in the negative control group, while the fluorescence intensity in the overexpression group was stronger. This reflects that Thbs4 could promote sciatic nerve axonal regeneration.

To explore the effects of Thbs4 on functional recovery after sciatic nerve injury, we performed footprint/Catwalk analysis and assessed thermal hyperalgesia and mechanical allodynia. The statistical analysis of SFI showed that the sciatic nerve functional recovery of rats after the overexpression of Thbs4 was better than that of the negative control group, while the recovery of rats after knockdown was worse (Figure 5G,H). The results for thermal hyperalgesia showed that the withdrawal time was longer in the knockdown group compared with the negative control group, while it was shorter in the overexpression group (Figure 5I,J). The results for mechanical allodynia showed that the foot withdrawal threshold was higher in the knockdown group than in the control group, while the threshold was lower in the overexpression group (Figure 5K,L). The above data indicate that Thbs4 promotes neurological function recovery.

### 3.6. Effects of Thbs4 on Myelin Sheath and Target Muscle

**Figure 6:**
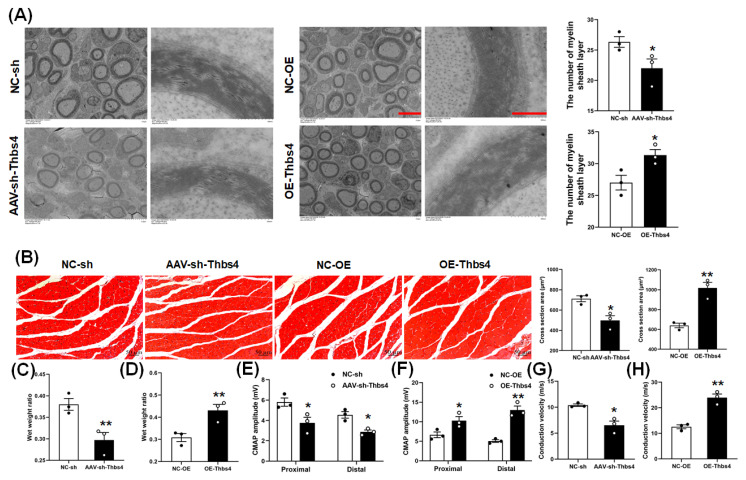
Effects of in vivo knockdown or overexpression of Thbs4 on sciatic nerve regeneration. (**A**) Transmission electron microscope (TEM) observation of myelin sheath of sciatic nerve (injured nerve) 8 weeks after knockdown or overexpression of Thbs4. Left bar = 5 μm and right bar = 500 nm. (**B**) H&E staining of gastrocnemius muscle (injured side) and cross-section area statistics of muscle fiber 8 weeks after knockdown or overexpression of Thbs4. (**C**,**D**) show the wet weight ratio of gastrocnemius muscle of rats after knockdown and overexpression of Thbs4 at 8 weeks, respectively. (**E**,**F**) show the CMAP amplitude of proximal and distal sciatic nerve 8 weeks after knockdown and expression of Thbs4, respectively. (**G**,**H**) show the conduction velocity of CMAP 8 weeks after knockdown and overexpression of Thbs4, respectively. NC-sh or NC-OE was used as negative control. *n* = 3, versus negative control, * *p* < 0.05 and ** *p* < 0.01. sh, short hairpin RNA. NC-sh, negative control of shRNA. AAV, adeno-associated virus. NC-OE, negative control of overexpressed AAV. Thbs4, thrombospondin-4. CMAP, compound muscle action potential.

A TEM was used to observe the myelin sheath of a regenerated sciatic nerve. The numbers of myelin sheath layers in regenerated sciatic nerves in the knockdown group was lower than that in the control group, while the number was higher in the overexpression group than in the control group (Figure 6A). This indicates that Thbs4 could promote myelination after injury.

Following H&E staining of gastrocnemius muscle, a cross-section area of muscle fibers was obtained via statistical analysis. This area of target muscle in the knockdown group was smaller than that in the control group, while the area in the overexpression group was larger than that in the control group (Figure 6B). The results for the gastrocnemius muscle wet weight ratio showed that the value in the knockdown group was lower, while the value in the overexpression group was higher than that in the negative control (Figure 6C,D). These data show that Thbs4 could alleviate muscle atrophy induced by sciatic nerve injury.

The amplitude of CMAP of sciatic nerve in both the proximal and distal ends in the knockdown group was lower than that in the control group, and the conduction velocity was also significantly lower than that of control group (Figure 6E,G). However, the CMAP amplitude and nerve conduction velocity in the overexpression group were higher than those in the control group (Figure 6F,H). The data also reflect that Thbs4 improved neurological recovery after injury.

### 3.7. Effects of Thbs4 on Signal Pathway Factors

**Figure 7:**
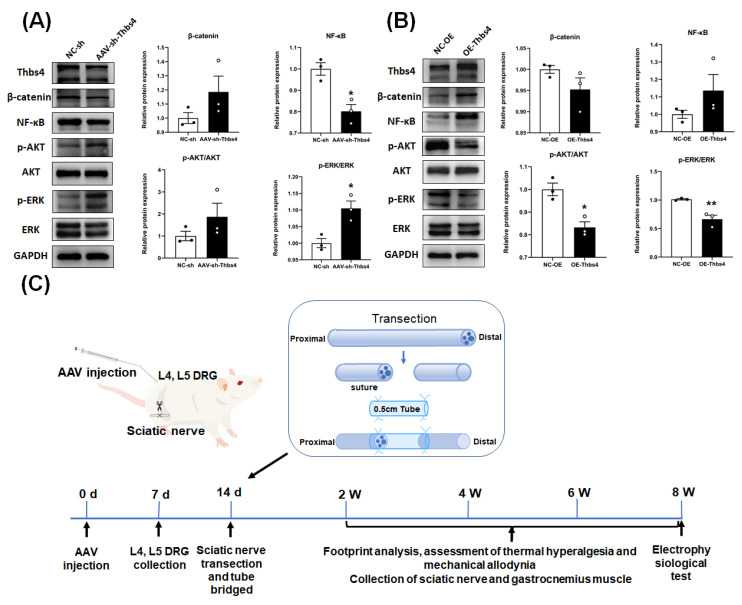
Effects of in vivo knockdown or overexpression of Thbs4 on signal pathway factors. (**A**,**B**) show Western blot used to detect the expressions of signal pathway factor after knockdown and overexpression of Thbs4, respectively. (**C**) Schematic diagram of sciatic nerve transection model. GADPH was used as internal control group, and NC-sh or NC-OE was used as negative control. *n* = 3, versus negative control, * *p* < 0.05 and ** *p* < 0.01. sh, short hairpin RNA. NC-sh, negative control of shRNA. AAV, adeno-associated virus. NC-OE, negative control of overexpressed AAV. Thbs4, thrombospondin-4. NF-κB, nuclear factor-κB. AKT, protein kinase B. ERK, extracellular regulated protein kinase. GAPDH, glyceraldehyde-3-phosphate dehydrogenase. qRT-PCR, real-time quantitative PCR.

A Western blot showed that p-ERK/ERK was significantly upregulated after the knockdown of Thbs4 in DRG tissues compared with the control group, while NF-κB was downregulated (Figure 7A). In the Thbs4 overexpression group, the p-ERK/ERK and p-AKT/AKT pathway were significantly downregulated (Figure 7B). This indicates that the ERK and NF-κB pathways may be involved in regulating DRG neuron functions by Thbs4.

## 4. Discussion

Peripheral nerve injury, which occurs outside the central nervous system, often leads to sensory disturbances, motor impairments, and nutritional imbalances in the affected area. This remains a significant challenge in regenerative medicine. Thbs4, a member of the thrombospondin protein family, is known for its roles in cell–matrix and cell–cell interactions [13,28]. Previous studies have highlighted its importance in cell migration [29], angiogenesis [30], and neurogenesis [31]. Our prior research using gene and protein chip technologies identified significant expression changes in Thbs4, NGF, and p53 during WD following sciatic nerve injury in rats, suggesting Thbs4’s potential role in peripheral nerve repair [24]. Despite extensive research on Thbs4 in cancer, angiogenesis, and cardiovascular diseases, its role in the nervous system, particularly in DRG neurons, remains understudied. This study explores the functional effects and mechanisms of Thbs4 in peripheral nerve repair through in vitro and in vivo manipulations of Thbs4 expression.

Firstly, we established a time-course model of sciatic nerve transection injury and found that Thbs4 expression was increased in DRG after sciatic nerve injury, reaching a peak on day 7 and then decreasing slowly. This suggests that Thbs4 may be involved in regulating peripheral nerve regeneration. After sciatic nerve injury, increased Thbs4 expression may promote neurite outgrowth. Then we detected the subcellular localization of Thbs4 expression in DRG. The results showed that Thbs4 was distributed in the cytoplasm and intercellular space of DRG tissues, mainly in the cytoplasm of DRG neurons. To further investigate the effects of Thbs4 expression on DRG neuronal function, we cultured primary DRG neuron cells transfected with siRNA to knock down Thbs4 or infected the cells with AAV to overexpress the gene. Then, cell viability, apoptosis, and neurite outgrowth were analyzed. The results showed that knockdown of Thbs4 significantly reduced cell vitality, DRG neuron neurite outgrowth was slower than that in the control group, and the apoptosis rate was relatively higher than that in the control group. However, the overexpression of Thbs4 tended to increase cell viability, neurite outgrowth was faster than that in the control group, and its apoptosis rate was also significantly lower than that in the control group. Therefore, we believe that Thbs4 impacts the biological function of DRG neuron cells cultured in vitro.

To further explore how Thbs4 regulates these functional changes, we selected factors closely related to apoptosis, neurite outgrowth, and pro-inflammatory responses. qRT-PCR analysis showed that the expression of NF200, PKCα, bFGF, and GAP43 decreased significantly after Thbs4 knockdown, but increased significantly after Thbs4 overexpression. Based on research reports, we know that NF200, as the main skeletal protein of mature neurons, plays an important role in the regeneration of neurons after injury in addition to its role as a scaffold [32]. PKCα, as one of the PKC isoforms, has been found to be widely distributed in various tissues and plays an important role in cell differentiation [33]. PKCα, as one of PKC isoforms, has been detected to be widely distributed in various tissues and plays an important role in cell differentiation [34]. Furthermore, some experiments suggest that PKCα may be involved in the differentiation of mouse embryonic stem cells into neuron-like cells, suggesting that the number of neurons may be closely related to the expression of PKCα [35]. Similarly, during H_2_O_2_-induced neuronal apoptosis, PKCα signaling can be activated to protect neurons and improve survival [36]. bFGF has been shown to significantly promote growth in a variety of cells, including neuronal survival, and is a growth-promoting polypeptide [37]. In a study of spinal cord injury in rats, bFGF expression increased significantly within one week of injury, suggesting a protective role in injury repair [38]. GAP43 plays an important role in guiding axon growth and regulating synaptic reconstruction [39]. Some studies have shown that GAP43 leads to neurons growing new terminals without additional nutrients, so changes in GAP43 are considered to be a marker of nerve repair [40]. Our results indicate that changes in Thbs4 expression at the mRNA level significantly affect the expression of factors associated with neuronal survival and growth. This suggests that Thbs4 may influence DRG neuronal function by regulating the expressions of these factors for peripheral nerve repair after injury.

How does Thbs4 influence neural regeneration through signaling pathways? We continued to select pathway proteins related to nerve regeneration based on the literature. We extracted proteins from cultured DRG neurons treated with Thbs4 knockdown and overexpression in vitro, as well as proteins from L4 and L5 tissues after an intrathecal injection inducing Thbs4 knockdown and overexpression in vivo. We then combined in vitro and in vivo data from Western blot analysis. These results showed that the level of NF-κB protein expression increased significantly when Thbs4 was overexpressed, while the reverse trend was observed after Thbs4 knockdown. The level of p-ERK/ERK protein expression decreased significantly after Thbs4 overexpression, while the reverse trend was observed after Thbs4 knockdown. It was reported that NF-κB, as a universally expressed complex transcription factor, is involved in regulating the expression of many genes, involved in cell survival, tumor development, and other life activities [41]. Some experimental studies have found that NF-κB is positive in the regeneration process of retinal neuroepithelial cells after injury, and thus it may be involved in axonal regeneration after injury [42]. In addition, some researchers have found that NF-κB may be involved in axon growth in neural progenitor cells. The inactivation of NF-κB leads to atrophy of the dentate gyrus (DG) region and prevents new neurons from developing [43].

ERK is an evolutionarily conserved serine/threonine protein kinase, which can transform stimulation into an intracellular response by regulating gene expression and participate in regulating cell survival [44]. There is evidence that ERK activation in the spinal cord is caused by various noxious stimuli, and ERK is activated in many places, such as the dorsal horn of the spinal cord when the peripheral body and viscera are stimulated or inflamed [45,46].

In addition, Thbs4 has been shown to promote neuronal differentiation in NG2 cells. Overexpression of Thbs4 increases the expression of neuronal differentiation markers, such as Tuj1 and NeuN, while strongly inhibiting the ERK signaling pathway. Combined with the above findings, our results suggest that Thbs4 may influence neural regeneration by regulating the NF-κB and ERK signaling pathways.

Next, we established an animal model via the intrathecal injection of AAV to knockdown or overexpress Thbs4, and SCG10 fluorescence staining was performed to detect the regenerated sciatic nerve [47,48]. The results showed that after the knockdown of Thbs4, fluorescence intensity was weaker than that in the control group, while the SCG10 fluorescence intensity of the overexpression group was stronger than that of the control group. Therefore, the overexpression of Thbs4 in vivo can promote sciatic nerve axon regeneration.

Subsequently, in order to study the recovery of peripheral nerve function after the knockdown or overexpression of Thbs4, we performed thermal pain, mechanical pain, gait, and electrophysiological tests at different time points, measured the wet weight ratio of gastrocnemius muscle, and observed the morphology of the regenerated nerve under an electron microscope. The recovery of sensory function in rats was measured to thermal and mechanical pain. The results showed that after the knockdown of Thbs4, the withdrawal time was longer than that in the control group, and the response to stimulation was stronger than that in the control group. After overexpression of Thbs4, the withdrawal time was shorter than that in control group, and the response to stimulation was weaker than that in control group, indicating that Thbs4 had a positive effect on the recovery of sensory function in rats. Gait analysis was used to detect the recovery of motor function in rats. The results showed that after knockdown with Thbs4, the recovery of sciatic nerve in rats was worse than that in control group, and after overexpression of Thbs4, the recovery of sciatic nerve in rats was better than that in the control group, indicating that Thbs4 also promoted the recovery of motor function. In addition, the wet weight ratio and H&E staining of gastrocnemius muscle at different time points showed that the atrophy of gastrocnemius muscle was more serious in the knockdown group than in the control group, and the atrophy of gastrocnemius muscle less significantly in the overexpression group than in the control group. Electrophysiological tests were performed at 8 weeks to determine the extent of nerve conduction recovery. The CMAP amplitude and nerve conduction velocity in Thbs4 overexpression group were higher than those in the control group. The results also showed that Thbs4 promoted nerve conduction recovery. In order to further examine the morphological recovery of the regenerated sciatic nerve, we observed the myelin sheath of the sciatic nerve and photographed its distal end under a transmission electron microscope. The results showed that after the knockdown of Thbs4, the number of myelin layers in the regenerated sciatic nerve in rats was lower than that in the control group, while after overexpression of Thbs4, the number of myelin layers in the regenerated sciatic nerve in rats was higher than that in the control group, further indicating that Thbs4 promoted sciatic nerve recovery.

In summary, Thbs4 expression is upregulated in DRG tissues after sciatic nerve injury. Thbs4 promotes axonal regeneration and inhibits neurons apoptosis, playing a positive regulatory role in axonal regeneration and functional recovery after sciatic nerve injury. It may regulate DRG neuronal function at the mRNA level by modulating the expression of NF200, PKCα, bFGF, and GAP43. The ERK and NF-κB signaling pathways may be involved in the regulation of DRG neurons and the promotion of nerve repair and regeneration. In vivo functional tests showed that the overexpression of Thbs4 significantly promoted the recovery of sensory and motor function after sciatic nerve injury, highlighting its importance in peripheral nerve repair. Our study provides a detailed investigation of the mechanisms by which Thbs4 functions in peripheral nerve injury repair both in vivo and in vitro, offering new insights and potential directions for the repair of peripheral nerve injuries.

## 5. Conclusions

Thbs4 mainly exists in the DRG cell membrane and stroma and is also distributed in the intercellular space. Thbs4 expression is upregulated in DRG tissues following sciatic nerve injury. In vitro studies demonstrated that the overexpression of Thbs4 significantly enhanced axonal regeneration and reduced neuronal apoptosis, whereas Thbs4 knockdown had the opposite effects. Similarly, in vivo experiments revealed that the overexpression of Thbs4 accelerated sciatic nerve regeneration and improved the recovery of motor and sensory function following sciatic nerve injury, whereas Thbs4 knockdown led to the opposite outcomes. The results of in vitro and in vivo experiments showed that the expression of the NF-κB and ERK changed significantly after the knockdown and overexpression of Thbs4, suggesting that the ERK and NF-κB signaling pathways may be involved in regulating the function of DRG neurons and promoting nerve repair and regeneration by Thbs4.

## Figures and Tables

**Table 1:** qRT-PCR primer sequences.

Gene	Sequence
Thbs4	F: 5′ GTGCATTGAGGAACGGCAAG 3′ R: 5′ CATCCCCGTCAGTGTCCTTCC 3′
Bcl2	F: 5′ GCAGAGATGTCCAGTCAGC 3′ R: 5′ CCCACCGAACTCAAAGAAGG 3′
Bax	F: 5′ TGCAGAGGATGATTGCTGAC 3′ R: 5′ GATCAGCTCGGGCACTTTAG 3′
NF200	F: 5′ GTCAGAGGAGTGGTTCCGAG 3′ R: 5′ GCCGCCGGTACTCAGTTATC 3′
PKCα	F: 5′ GAACACATGATGGACGGGGTCACGAC 3′ R: 5′ CGCTTGGCAGGGTGTTTGGTCATA 3′
bFGF	F: 5′ CCCGCACCCTATCCCTTCACAGC 3′ R: 5′ CACAACGACCAGCCTTCCACCCAAA 3′
AKT2	F: 5′ CCGGTGAACTCTGACCCTTG 3′ R: 5′ GGCCGCAGCGTCTTCAT 3′
TNFα	F: 5′ ATGGGCTCCCTCTCATCAGT 3′ R: 5′ GCTTGGTGGTTTGCTACGAC 3′
IL-1β	F: 5′ GACCTGTTCTTTGAGGCTGAC 3′ R: 5′ TCCATCTTCTTCTTTGGGTATTGTT 3′
GAP43	F: 5′ TGCTGTGCTGTATGAGAAGAAC 3′ R: 5′ TTGGTTGCAGCCTTATGAGC 3′
GAPDH	F: 5′ TGGAGTCTACTGGCGTCTT 3′ R: 5′ TGTCATATTTCTCGTGGTTCA 3′

**Table 2:** The used antibodies.

Antibody	Sources	Catalogue Number
Thbs4	Santa Cruz	Sc-7657-R
GAPDH	Proteintech	60004-1
β-catenin	Cell signaling	8480T
NF-κB	Cell signaling	8242T
ERK	Abcam	ab201015
p-ERK	Santa Cruz	Sc-7383
AKT	Cell signaling	4691T
p-AKT	Cell signaling	4060T
β-Tubulin III	Abcam	ab78078
SCG10	Novus	NBP1-4946
NeuN	Abcam	Ab177487
Goat Anti-Mouse IgG HRP	Abways	AB0102
Goat Anti-Rabbit IgG HRP	Abways	AB0101
Cy3-conjugated goat anti-Rabbit	Proteintech	SA00009-2
CoraLite488-conjugated goat anti-Rabbit	Proteintech	SA00013-2
CoraLite488-conjugated goat anti-Mouse	Proteintech	SA00013-1

**Table 3:** Thbs4-siRNA target sequences.

Product Number	Product Name	Target Sequence
siG150605090149	si-r-Thbs4_001	CCATCCTCCGTTACCTAAA
siG150605090215	si-r-Thbs4_002	GGATGAGTGTAAATACCAT
siG150605090241	si-r-Thbs4_003	GGACCAGAGGAACACTGAT

## Data Availability

All data generated or analyzed during this study are included in this published article.

## References

[1] O’Brien A.L., West J.M., Saffari T.M., Nguyen M., Moore A.M. (2022). Promoting Nerve Regeneration: Electrical Stimulation, Gene Therapy, and Beyond. Physiology.

[2] Min Q., Parkinson D.B., Dun X.P. (2021). Migrating Schwann cells direct axon regeneration within the peripheral nerve bridge. Glia.

[3] Wu S., Shen W., Ge X., Ao F., Zheng Y., Wang Y., Jia X., Mao Y., Luo Y. (2023). Advances in Large Gap Peripheral Nerve Injury Repair and Regeneration with Bridging Nerve Guidance Conduits. Macromol. Biosci..

[4] Mahar M., Cavalli V. (2018). Intrinsic mechanisms of neuronal axon regeneration. Nat. Rev. Neurosci..

[5] Wu J., Lu B., Yang R., Chen Y., Chen X., Li Y. (2021). EphB2 knockdown decreases the formation of astroglial-fibrotic scars to promote nerve regeneration after spinal cord injury in rats. CNS Neurosci. Ther..

[6] Krishnan A., Verge V.M.K., Zochodne D.W. (2024). Hallmarks of peripheral nerve injury and regeneration. Handb. Clin. Neurol..

[7] Bolandghamat S., Behnam-Rassouli M. (2020). Recent Findings on the Effects of Pharmacological Agents on the Nerve Regeneration after Peripheral Nerve Injury. Curr. Neuropharmacol..

[8] Yin Q., Kemp G.J., Yu L.G., Wagstaff S.C., Frostick S.P. (2015). Neurotrophin-4 delivered by fibrin glue promotes peripheral nerve regeneration. Muscle Nerve.

[9] Shen J., Sun Y., Liu X., Chai Y., Wang C., Xu J. (2024). Nerve Regeneration Potential of Antioxidant-Modified Black Phosphorus Quantum Dots in Peripheral Nerve Injury. ACS Nano.

[10] Zhang Y., Zhao Q., Chen Q., Xu L., Yi S. (2023). Transcriptional Control of Peripheral Nerve Regeneration. Mol. Neurobiol..

[11] Genaro K., Luo Z.D. (2024). Pathophysiological roles of thrombospondin-4 in disease development. Semin. Cell Dev. Biol..

[12] Muppala S., Xiao R., Gajeton J., Krukovets I., Verbovetskiy D., Stenina-Adognravi O. (2021). Thrombospondin-4 mediates hyperglycemia- and TGF-beta-induced inflammation in breast cancer. Int. J. Cancer.

[13] Dobelmann V., Roos A., Hentschel A., Della Marina A., Leo M., Schmitt L.I., Maggi L., Schara-Schmidt U., Hagenacker T., Ruck T. (2024). Thrombospondin-4 as potential cerebrospinal fluid biomarker for therapy response in pediatric spinal muscular atrophy. J. Neurol..

[14] Wu Y., Yang M., Xu X., Gao Y., Li X., Li Y., Su S., Xie X., Yang Z., Ke C. (2024). Thrombospondin 4, a mediator and candidate indicator of pain. Eur. J. Cell Biol..

[15] Stenina-Adognravi O., Plow E.F. (2019). Thrombospondin-4 in tissue remodeling. Matrix Biol..

[16] Yang H.J., Ma S.P., Ju F., Zhang Y.P., Li Z.C., Zhang B.B., Lian J.J., Wang L., Cheng B.F., Wang M. (2016). Thrombospondin-4 Promotes Neuronal Differentiation of NG2 Cells via the ERK/MAPK Pathway. J. Mol. Neurosci..

[17] Zierfuss B., Hobaus C., Herz C.T., Pesau G., Koppensteiner R., Schernthaner G.H. (2020). Thrombospondin-4 increases with the severity of peripheral arterial disease and is associated with diabetes. Heart Vessels.

[18] Zeng H., Lan B., Li B., Xie H., Zhao E., Liu X., Xue X., Sun J., Su L., Zhang Y. (2024). The role and mechanism of thrombospondin-4 in pulmonary arterial hypertension associated with congenital heart disease. Respir. Res..

[19] Guo Y., Zhang Z., Wu H.E., Luo Z.D., Hogan Q.H., Pan B. (2017). Increased thrombospondin-4 after nerve injury mediates disruption of intracellular calcium signaling in primary sensory neurons. Neuropharmacology.

[20] Pan B., Yu H., Park J., Yu Y.P., Luo Z.D., Hogan Q.H. (2015). Painful nerve injury upregulates thrombospondin-4 expression in dorsal root ganglia. J. Neurosci. Res..

[21] Jiang B.C., Liu T., Gao Y.J. (2020). Chemokines in chronic pain: Cellular and molecular mechanisms and therapeutic potential. Pharmacol. Ther..

[22] Park J.F., Yu Y.P., Gong N., Trinh V.N., Luo Z.D. (2018). The EGF-LIKE domain of thrombospondin-4 is a key determinant in the development of pain states due to increased excitatory synaptogenesis. J. Biol. Chem..

[23] Yu Y.P., Gong N., Kweon T.D., Vo B., Luo Z.D. (2018). Gabapentin prevents synaptogenesis between sensory and spinal cord neurons induced by thrombospondin-4 acting on pre-synaptic Ca(v)alpha(2)delta(1) subunits and involving T-type Ca(2+) channels. Br. J. Pharmacol..

[24] Yao D., Li M., Shen D., Ding F., Lu S., Zhao Q., Gu X. (2012). Gene expression profiling of the rat sciatic nerve in early Wallerian degeneration after injury. Neural Regen. Res..

[25] Percie du Sert N., Hurst V., Ahluwalia A., Alam S., Avey M.T., Baker M., Browne W.J., Clark A., Cuthill I.C., Dirnagl U. (2020). The ARRIVE guidelines 2.0: Updated guidelines for reporting animal research. PLoS Biol..

[26] Feng Y.M., Shao J., Cai M., Zhou Y.Y., Yao Y., Qian J.X., Ding Z.H., Jiang M.R., Yao D.B. (2023). Long noncoding RNA H19 regulates degeneration and regeneration of injured peripheral nerves. Neural Regen. Res..

[27] Zhou Y., Yao Y., Feng Y., Qiu Z., Luo S., Shi X., Gu D., Jiang M., Cai M., Yao D. (2024). Fas ligand regulate nerve injury and repair by affecting AKT, beta-catenin, and NF-kappaB pathways. IBRO Neurosci. Rep..

[28] Zaren P., Gawlik K.I. (2024). Thrombospondin-4 deletion does not exacerbate muscular dystrophy in beta-sarcoglycan-deficient and laminin alpha2 chain-deficient mice. Sci. Rep..

[29] Chou K.Y., Chang A.C., Ho C.Y., Tsai T.F., Chen H.E., Chen P.C., Hwang T.I. (2021). Thrombospondin-4 promotes bladder cancer cell migration and invasion via MMP2 production. J. Cell. Mol. Med..

[30] Zhang Q., Zhang Z., Xiu Y., Zou T., Quan Y. (2025). Sodium ferulate attenuates ischaemic stroke by mediating the upregulation of thrombospondin-4 expression and combined treatment with bone marrow mesenchymal stem cells. Exp. Neurol..

[31] Zhao T., Wang Z., Zhu T., Xie R., Zhu J. (2020). Downregulation of Thbs4 caused by neurogenic niche changes promotes neuronal regeneration after traumatic brain injury. Neurol. Res..

[32] Ye Z., Zheng Y., Li N., Zhang H., Li Q., Wang X. (2024). Repair of spinal cord injury by bone marrow mesenchymal stem cell-derived exosomes: A systematic review and meta-analysis based on rat models. Front. Mol. Neurosci..

[33] Yu J., Xiang Y., Gao Y., Chang S., Kong R., Lv X., Yu J., Jin Y., Li C., Ma Y. (2024). PKCalpha inhibitors promote breast cancer immune evasion by maintaining PD-L1 stability. Acta Pharm. Sin. B.

[34] Singh R.K., Kumar S., Kumar S., Shukla A., Kumar N., Patel A.K., Yadav L.K., Kaushalendra, Antiwal M., Acharya A. (2023). Potential implications of protein kinase Calpha in pathophysiological conditions and therapeutic interventions. Life Sci..

[35] Godoy-Parejo C., Deng C., Xu J., Zhang Z., Ren Z., Ai N., Liu W., Ge W., Deng C., Xu X. (2023). Protein Kinase C Modulation Determines the Mesoderm/Extraembryonic Fate Under BMP4 Induction From Human Pluripotent Stem Cells. Stem Cells.

[36] Su L.Y., Jiao L., Liu Q., Qiao X., Xie T., Ma Z., Xu M., Ye M.S., Yang L.X., Chen C. (2024). S-nitrosoglutathione reductase alleviates morphine analgesic tolerance by restricting PKCalpha S-nitrosation. Redox Biol..

[37] Li H., Gan X., Pan L., Zhang Y., Hu X., Wang Z. (2022). EGF/bFGF promotes survival, migration and differentiation into neurons of GFP-labeled rhesus monkey neural stem cells xenografted into the rat brain. Biochem. Biophys. Res. Commun..

[38] Chen X., Wang B., Zhou Y., Wu X., Du A., Al Mamun A., Xu Y., Wang S., Jiang C., Xie L. (2024). Poly (Betulinic Acid) Nanoparticles Loaded with bFGF Improve Functional Recovery After Spinal Cord Injury. Adv. Healthc. Mater..

[39] Lisek M., Tomczak J., Boczek T., Zylinska L. (2024). Calcium-Associated Proteins in Neuroregeneration. Biomolecules.

[40] Xie X., Wan J., Zheng X., Pan W., Yuan J., Hu B., Feng M., Liu Z., Cai S. (2022). Synergistic effects of epigallocatechin gallate and l-theanine in nerve repair and regeneration by anti-amyloid damage, promoting metabolism, and nourishing nerve cells. Front. Nutr..

[41] Williams L.M., Gilmore T.D. (2020). Looking Down on NF-kappaB. Mol. Cell. Biol..

[42] Zhou L., Kong G., Palmisano I., Cencioni M.T., Danzi M., De Virgiliis F., Chadwick J.S., Crawford G., Yu Z., De Winter F. (2022). Reversible CD8 T cell-neuron cross-talk causes aging-dependent neuronal regenerative decline. Science.

[43] Kijima K., Ono G., Kobayakawa K., Saiwai H., Hara M., Yoshizaki S., Yokota K., Saito T., Tamaru T., Iura H. (2023). Zinc deficiency impairs axonal regeneration and functional recovery after spinal cord injury by modulating macrophage polarization via NF-kappaB pathway. Front. Immunol..

[44] Tanimura S., Takeda K. (2017). ERK signalling as a regulator of cell motility. J. Biochem..

[45] Booth L.A., Roberts J.L., Dent P. (2020). The role of cell signaling in the crosstalk between autophagy and apoptosis in the regulation of tumor cell survival in response to sorafenib and neratinib. Semin. Cancer Biol..

[46] Damasio M.P., Marchingo J.M., Spinelli L., Hukelmann J.L., Cantrell D.A., Howden A.J.M. (2021). Extracellular signal-regulated kinase (ERK) pathway control of CD8+ T cell differentiation. Biochem. J..

[47] Li Y., Tian Y., Pei X., Zheng P., Miao L., Li L., Luo C., Zhang P., Jiang B., Teng J. (2023). SCG10 is required for peripheral axon maintenance and regeneration in mice. J. Cell Sci..

[48] Baughn M.W., Melamed Z., Lopez-Erauskin J., Beccari M.S., Ling K., Zuberi A., Presa M., Gonzalo-Gil E., Maimon R., Vazquez-Sanchez S. (2023). Mechanism of STMN2 cryptic splice-polyadenylation and its correction for TDP-43 proteinopathies. Science.

